# Preferences of patients and physicians in the United States for relapsed/refractory follicular lymphoma treatments

**DOI:** 10.1002/cam4.70177

**Published:** 2024-10-12

**Authors:** Caitlin Thomas, Kevin Marsh, Myrto Trapali, Nicolas Krucien, Gavin Worth, Paul Cockrum, Debra Lycett

**Affiliations:** ^1^ Patient‐Centered Research, Evidera London UK; ^2^ Ipsen, Boulogne‐Billancourt Paris France; ^3^ Ipsen Cambridge Massachusetts USA

**Keywords:** discrete‐choice experiment, progression‐free survival, relapsed/refractory follicular lymphoma, relative attribute importance, shared decision‐making, treatment preference

## Abstract

**Background:**

Patients with follicular lymphoma (FL) often relapse or become refractory to treatment (R/R). While the R/R FL treatment landscape evolves, little is known about the priorities of patients and physicians. This discrete‐choice experiment (DCE) study assessed patients' and physicians' treatment preferences, and the trade‐offs they would be willing to make between efficacy, tolerability, and administration.

**Methods:**

An online survey was conducted in US‐based patients (≥18 years) with R/R FL and FL‐treating physicians. The DCE was informed by a targeted literature review, clinical data, expert oncologist input, and pilot interviews. Participants completed eight experimental choice tasks where they chose between two hypothetical treatment profiles defined by six attributes: progression‐free survival (PFS), administration/monitoring, risks of laboratory abnormalities requiring intervention, severe infections, diarrhea, and cytokine release syndrome (CRS). Relative attribute importance (RAI) and willingness to trade‐off between PFS and other attributes were estimated.

**Results:**

Two‐hundred patients (mean age 63.5 years; median three prior lines of therapy) and 151 FL‐treating physicians participated. Increasing PFS was most important for both groups, although it was relatively less important to patients than physicians (RAI 35.2% vs. 45.7%). Administration/monitoring was three times more important to patients than physicians (RAI 28.8% vs. 9.5%); patients preferred oral treatment and would be willing to tolerate a significant reduction in PFS for oral administration over weekly intravenous infusions. Avoiding CRS was less important to patients than to physicians (RAI 7.7% vs. 15.8%). Both groups would accept shorter PFS for reduced risks of side effects (especially of laboratory abnormalities for patients and of CRS for physicians).

**Conclusion:**

Although PFS was the most important attribute to patients and physicians, both would tolerate lower PFS for reduced side effects. Patients would also accept a substantial reduction in PFS for oral administration. Differences between the preferences/priorities of patients and physicians highlight the importance of shared decision‐making.

## 1 INTRODUCTION

Follicular lymphoma (FL) is the second most common subtype of non‐Hodgkin lymphoma worldwide and often presents as a painless, slowly progressive adenopathy.^1^, ^2^, ^3^ Prognosis of FL has improved in recent years^3^ and patients' survival may exceed 20 years, with approximately 80% surviving >10 years.^4^, ^5^ However, most patients present with advanced disease and often develop relapsed or refractory (R/R) FL.^1^, ^2^, ^6^ Many patients undergo several treatment lines, each with successively shorter progression‐free survival (PFS).^7^ The patient's disease journey and treatment history will affect individual treatment decisions.^8^

The development of more targeted treatments has significantly expanded the therapy landscape for R/R FL.^1^ Current treatment options in later lines include chemotherapy, rituximab with lenalidomide or as a single agent, tazemetostat, chimeric antigen receptor T‐cell (CAR‐T) therapy, and bispecific antibodies.^1^, ^2^, ^4^, ^8^ Despite several therapies being available for R/R FL, existing treatments differ in their efficacy, safety, and mode of administration.^3^ Differences in these treatment attributes should be carefully considered when making treatment choices,^3^, ^4^ as they might impact patients' preferences or the physicians' decision to prescribe a treatment.

Physicians' considerations of treatments are influenced by factors including disease stage, prior therapy, safety concerns, and comorbidities.^3^ Additionally, engaging patients in the decision‐making process is increasingly promoted.^9^, ^10^ However, there is growing appreciation that patients and physicians might differ in their treatment perceptions, goals, preferences, and trade‐offs.^9^, ^10^, ^11^ Thus, as part of the shared decision‐making, it is also important to understand how both physicians and patients perceive the benefits and risks of treatments and to determine the trade‐offs they would be willing to make, as treatments with higher efficacy often come with additional tolerability concerns.^4^, ^10^, ^12^, ^13^

Preferences for different treatment attributes can be elicited using discrete‐choice experiments (DCEs), in which participants are presented with tasks where they are asked to select a preferred treatment option from a set of hypothetical alternatives.^14^, ^15^ DCEs are increasingly used to assess to what extent participants value each treatment attribute and to estimate the trade‐offs they would be willing to make.^14^, ^15^ Research in Canada found that patients with R/R FL required a 0.6‐year increase in PFS to accept the toxicity associated with autologous stem cell transplant (relative to chemotherapy).^13^ A recent multi‐country study found that physicians also made trade‐offs between efficacy and safety and that they placed more value on PFS than on overall survival (OS) or side effects.^16^ However, in R/R FL, the preference literature is very limited, and little is known about how the preferences and trade‐offs of patients and physicians might vary.

The present DCE study assessed the treatment preferences of US‐based patients with R/R FL and US‐based physicians currently treating patients with FL, including the relative importance that patients and physicians placed on the different attributes, and the trade‐offs that they would be willing to make between attributes. This study included the largest sample of patients with R/R FL and the largest sample of US‐based physicians to date.

## 2 METHODS

### 2.1 Overall study design

Adult patients (≥18 years) with R/R FL and physicians currently treating FL completed an online survey between September and November 2021 which included a DCE to understand treatment preferences of both participant groups. In the DCE, participants completed a series of choice tasks where they selected between two hypothetical treatment profiles described by varying levels of key treatment attributes. The participants' preference data obtained were used to infer the relative importance that participants place on each attribute.^14^, ^17^ Treatment attributes and levels were informed by a targeted literature review, extracted clinical data, and input from an expert oncologist and the Lymphoma Research Foundation. To confirm the DCE was feasible, robust, and clear, an initial version was developed and pilot‐tested in cognitive interviews with five patients with R/R FL and five FL‐treating physicians. Pilot interviews lasted 60 min and were conducted in July 2021. The study was approved by Salus institutional review board (formerly Ethical & Independent; study number: 21809‐01) in June 2021 and an amendment was approved in August 2021.

### 2.2 Participants

Participants were recruited by a third‐party vendor, Global Perspectives, with patients recruited via physician referrals, and physicians identified through healthcare provider databases and panels. The vendor sent invitations to potential participants, who were screened via telephone for the pilot interviews or with a self‐administered screening form for the main online survey.

To be eligible, patients had to be ≥18 years of age and report a diagnosis of FL and failure on ≥2 lines of therapy (based on feedback when asked about their treatment history). Physicians were required to be treating FL and to have treated FL in the previous year. Both patients and physicians had to be US‐based, able to read and understand English, provide informed consent, for interviews they had to consent to be audio‐recorded, and not have a hearing impairment that would interfere with their participation. Participants received compensation, in line with fair market value, for their time.

### 2.3 Attribute and level development

#### 2.3.1 Targeted literature review and clinical data extraction

A targeted literature review conducted between November and December 2020 identified potential attributes for inclusion in the DCE. Due to the limited preference research in FL, searches included populations with non‐Hodgkin lymphoma, nodular lymphoma, and FL. From the 62 papers identified, six (three qualitative and three quantitative preference studies^13^, ^18^, ^19^, ^20^, ^21^, ^22^) were included for data extraction. Efficacy and safety data as well as administration/monitoring procedures required were extracted from these six studies, and key differentiators across treatments were identified. The treatments of interest included commonly used therapies and those in development for R/R FL, including monotherapies (axicabtagene ciloleucel, bendamustine, copanlisib, duvelisib, idelalisib, mosunetuzumab, parsaclisib, rituximab, tazemetostat, tisagenlecleucel, umbralisib, and zandelisib) and combination therapies (bendamustine‐rituximab, lenalidomide plus rituximab followed by lenalidomide, obinutuzumab plus bendamustine, venetoclax plus bendamustine‐rituximab, and venetoclax plus rituximab).

#### 2.3.2 Attribute and level selection

The selection of attributes for inclusion in the DCE was informed by the targeted literature review and extracted clinical data. Potential attributes were further defined through discussions with clinical experts, including an external expert oncologist. Due to the long OS in R/R FL (10‐year OS ~80%) and given that treatment‐related mortality is expected to be low,^4^, ^5^ OS was not included as a study attribute that varied. However, for a complete picture, participants were informed that the duration of OS would be 10 years across all treatment options. The six attributes included in the final DCE were PFS, administration/monitoring, and the risks of four side effects: cytokine release syndrome (CRS), severe infections, diarrhea, and laboratory abnormalities requiring intervention. The level ranges shown for each attribute were selected to cover the breadth of outcomes across the treatments of interest (i.e., to include the minimum and maximum values identified in the clinical data extraction per attribute). The treatment alternatives that participants were asked to choose between in the DCE were characterized by these pre‐defined attributes, with varying levels (Table 1).

**TABLE 1:** Treatment attributes and levels.

Attribute groups	Attributes (Physicians)	Attributes (Patients)	Levels
Administration and monitoring	Administration and monitoring	Administration and check‐ups	Oral pills daily, monitoring every 4 weeks Oral pills daily, monitoring every 6 weeks IV infusion every week, monitoring during infusion appointment IV infusion every month, monitoring during infusion appointment IV infusion every month and daily oral pill, monitoring during infusion appointment CAR‐T therapy. Takes 1 month—one‐time treatment. Inpatient in hospital for 7 days after treatment. Must stay near hospital for 4 weeks for monitoring
Benefit	PFS and OS^a^	Time without cancer growth and time alive	6 months PFS, 10 years OS 1 year and 4 months PFS, 10 years OS 2 years and 2 months PFS, 10 years OS 3 years PFS, 10 years OS
Side effects	Risk of diarrhea	Number of people who have diarrhea	15% (15 out of 100 people) 25% (25 out of 100 people) 50% (50 out of 100 people)
	Risk of severe infections	Number of people who have severe Infections	0% (0 out of 100 people) 10% (10 out of 100 people) 25% (25 out of 100 people)
	Risk of severe laboratory abnormalities requiring intervention	Number of people who have blood test results requiring intervention	0% (0 out of 100 people) 50% (50 out of 100 people) 100% (100 out of 100 people)
	CRS	Inflammation	Very high risk^b^ No risk

Abbreviations: CAR‐T, chimeric antigen receptor T‐cell; CRS, cytokine release syndrome; IV, intravenous; OS, overall survival; PFS, progression‐free survival.

^a^ Overall survival was fixed at 10 years for all treatment options in the survey.

^b^ Defined as 5% of people not having CRS, 80% having non‐severe CRS side effects, 10% having non‐fatal CRS side effects, and 5% having fatal CRS side effects.

### 2.4 DCE instrument design

**FIGURE 1:**
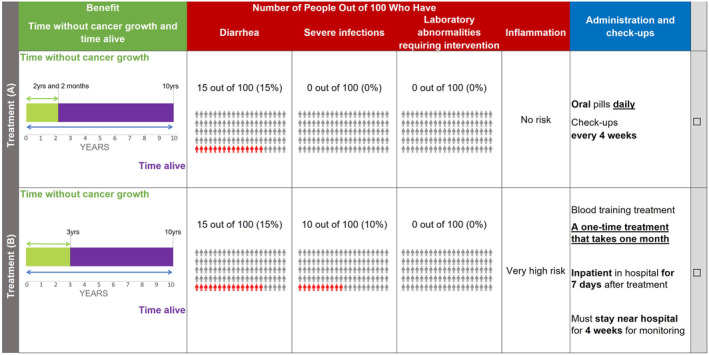
Example choice question. After selecting their preferred treatment option, participants were asked to answer a follow‐up opt‐out question. Question for patients: “If these treatments had been offered to you by your doctor, would you have (A) taken the treatment that you just chose above or (B) taken neither of the treatments?” Question for physicians: “If you had these treatments available to recommend to patients, would you have: (A) recommended the treatment that you chose above or (B) recommended neither of these treatments?”.

The DCE was generated with Ngene software (version 1.2, ChoiceMetrics, Australia) using a D‐efficient design with 24 tasks divided across three blocks; each participant was randomized to one of the blocks completing eight experimental choice tasks (Figure 1). Within each choice task, patients were first asked to choose their preferred treatment between two options, and then they were asked if they would take the treatment they chose or take neither of the treatments they were presented with, had these been offered by their doctor. Similarly, physicians were asked whether or not they would recommend the treatment they selected for a patient if these were actual options for a patient. After the eight experimental choice tasks, participants completed two additional choice tasks to assess the quality of the DCE data: a repeated‐choice task (the third choice task completed by the participant was repeated) to assess choice stability, and a dominance task (where one treatment option had superior efficacy and lower risks, and both treatment options had the same administration/monitoring) to assess engagement with the survey.

The same design was used for both patients and physicians. The order of the choice tasks within a block, the content of Treatment A and Treatment B, and the order of the attribute groups (and the individual attributes within the side effect group) were randomized to avoid introducing potential ordering, learning, and fatigue bias.^23^, ^24^ All randomizations were between participants.

Constraints on some attribute combinations were applied to ensure clinical credibility for the physician population. Hypothetical treatment profiles showing CAR‐T therapy administration were not shown with “No risk” of CRS or “0% (0 out of 100 people)” risk of severe lab abnormalities requiring intervention. To avoid attribute dominance, which is a risk in oncology DCEs, and thus increase the likelihood of trade‐offs, the maximum difference in PFS levels shown between each pair of treatments was limited to 1 year and 8 months.

### 2.5 Survey flow

In Section 1, participants were introduced to the DCE attributes. The attribute names and definitions were tailored to participants such that non‐clinical, lay terminology was used with patients (Table S1). In Section 2, participants completed the eight experimental choice tasks in the DCE as well as the two data quality tasks. In Section 3, participants were asked to complete a health literacy and numeracy questionnaire, as well as sociodemographic and clinical questionnaires.

### 2.6 Data analyses

All statistical tests were independent two‐sided z‐tests and used a significance level of 0.05. Analyses were conducted using R software (version 4.1.1).

#### 2.6.1 DCE data analysis

All DCE data were analyzed following within the random utility maximization framework theory. This theory assumes that respondents' preferences in a DCE task can be represented by a utility value, and that respondents always choose the treatment alternative that results in the highest utility (a measure of desirability) based on the included attributes and the importance placed on them. The utility function measures the effect of changes in each attribute, referred to as marginal utilities, on the probability of a treatment alternative being preferred.

The utility function was estimated using different logit models that made different assumptions about participants' preferences; these models were assessed to determine which were best at explaining (i.e. best statistical fit) patient and physician preferences. Patient heterogeneity was best represented with a discrete distribution of preferences, and three preference groups/classes provided the best representation of preferences.^25^ Physician heterogeneity was best represented with a continuous distribution of preferences, implemented within a mixed logit (MXL) model.^26^ This model assumes that individuals' preferences can be described with a statistical distribution (e.g. normally distributed) whose parameters (e.g., mean and variance) are to be estimated. The mean captures the average sensitivity to change in the attributes, while the variance measures the dispersion around this average effect and thus can be interpreted as a measure of preference heterogeneity.

#### 2.6.2 Relative attribute importance

Utility estimates were used to compute scores of the relative attribute importance (RAI) of each attribute over the level ranges included (which cover potential outcomes based on the extracted clinical data for the treatments of interest) to participants' treatment choices. RAI scores across attributes sum up to 100%. Differences in RAI scores of patients and physicians (ΔRAI) were compared with independent two‐sided z‐tests (with the null hypothesis being equality of scores).

#### 2.6.3 Willingness to trade‐off between attributes

Participants' willingness to make trade‐offs between efficacy and risk of side effects or administration/monitoring was calculated as the minimum acceptable improvement in PFS that they would require to tolerate/accept increased risks or inconveniences in administration/monitoring. Conversely, this can be interpreted as the lower level of PFS that participants would tolerate/accept for a treatment that had lower levels of risk or more convenient administration/monitoring.

## 3 RESULTS

### 3.1 Demographic and clinical characteristics

Of the 210 patients that were invited to take part in the study, 95% (*n* = 200) were interested and eligible, provided consent, and completed the main DCE survey. Of the 2000 physicians that were invited to take part, 8% (*n* = 151) were interested and eligible, provided consent, and completed the main DCE survey. Broad outreach for physicians was due to recruitment via physician panels for a target sample of *n* = 150 physicians.

The mean age of patients was 63.5 years (standard deviation [SD]: 5.6). Most patients were female (60%; *n* = 120), 40% (*n* = 80) were White, 21% (*n* = 41) were Black or African American, and 25% (*n* = 49) were Hispanic or Latino. Just over half of the patients (56%; *n* = 111) reported being in remission at the time of the survey. Patients had received a median of 3 (range: 2–4) prior lines of therapy, and the most common treatments received since FL diagnosis were rituximab monotherapy (98%; *n* = 195) and R‐CHOP (rituximab, cyclophosphamide, doxorubicin, vincristine, and prednisone) (77%; *n* = 154) (Table 2).

**TABLE 2:** Patient characteristics.

Characteristic	Patients (*N* = 200)^a^
Age (years), mean (SD)	63.5 (5.6)
Female sex at birth, *n* (%)	120 (60)
Racial background, *n* (%)	
White	80 (40)
Black or African American	41 (21)
Asian	14 (7)
Native Hawaiian or other Pacific Islander	17 (9)
American Indian or Alaska Native	11 (6)
Prefer not to say	37 (19)
Ethnic background, *n* (%)	
Hispanic or Latino	49 (25)
Not Hispanic or Latino	132 (66)
Prefer not to say	19 (10)
Number of initial lines of therapy, median (range)	3 (2–4)
Two lines of therapy, *n* (%)	65 (33)
Three lines of therapy, *n* (%)	124 (62)
Four lines of therapy, *n* (%)	11 (6)
Age at FL diagnosis, mean (SD)	59.4 (5.3)
Remission status, *n* (%)	
In remission	111 (56)
Not in remission	85 (43)
Prefer not to say	4 (2)
Treatments since initial FL diagnosis, *n* (%)^b^	
Rituximab monotherapy	195 (98)
R‐CHOP (rituximab, cyclophosphamide, doxorubicin, vincristine, and prednisone)	154 (77)
R‐CVP (rituximab, cyclophosphamide, vincristine, and prednisone)	106 (53)
R^2^ (lenalidomide and rituximab)	90 (45)
Obinutuzumab monotherapy	88 (44)
Idelalisib	39 (20)
BG (obinutuzumab and bendamustine)	33 (17)
Ibritumomab tiuxetan radioimmunotherapy	30 (15)
BR (bendamustine and rituximab)	20 (10)
Tazemetostat	20 (10)
G‐chemo (obinutuzumab and any chemotherapy)	17 (9)
Other	11 (6)
R‐ICE (rituximab, ifosfamide, carboplatin and etoposide)	1 (1)
Stem cell transplantation	1 (1)
Zandelisib	1 (1)

Abbreviations: FL, follicular lymphoma; SD, standard deviation.

^a^ Percentages may not sum 100 due to rounding.

^b^ Summarizes treatments reported by >40% of patient participants.

The physicians who participated in the study comprised 125 (83%) oncologists and 26 (17%) hematologists. They had treated FL for an average of 15 years and treated a mean of approximately 16 patients with FL per month (Table 3).

**TABLE 3:** Physician characteristics.

Characteristic	Physicians (*N* = 151)
Specialty, *n* (%)	
Hematologist	26 (17)
Oncologist	125 (83)
Years treating patients with lymphoma, mean (SD)	15.3 (7.6)
Number of patients with FL treated per month, mean (SD)	15.7 (18.8)

Abbreviation: SD, standard deviation.

### 3.2 Data quality

Ninety percent of patients (*n* = 179) and 87% of physicians (*n* = 132) selected the treatment option with higher benefits and lower risks in the dominance task, suggesting that the survey sufficiently engaged participants. Similarly, most patients (82%; *n* = 163) and physicians (78%; *n* = 118) provided consistent answers to the repeated‐choice question.

### 3.3 Patient and physician preferences for attributes of R/R FL treatments

**FIGURE 2:**
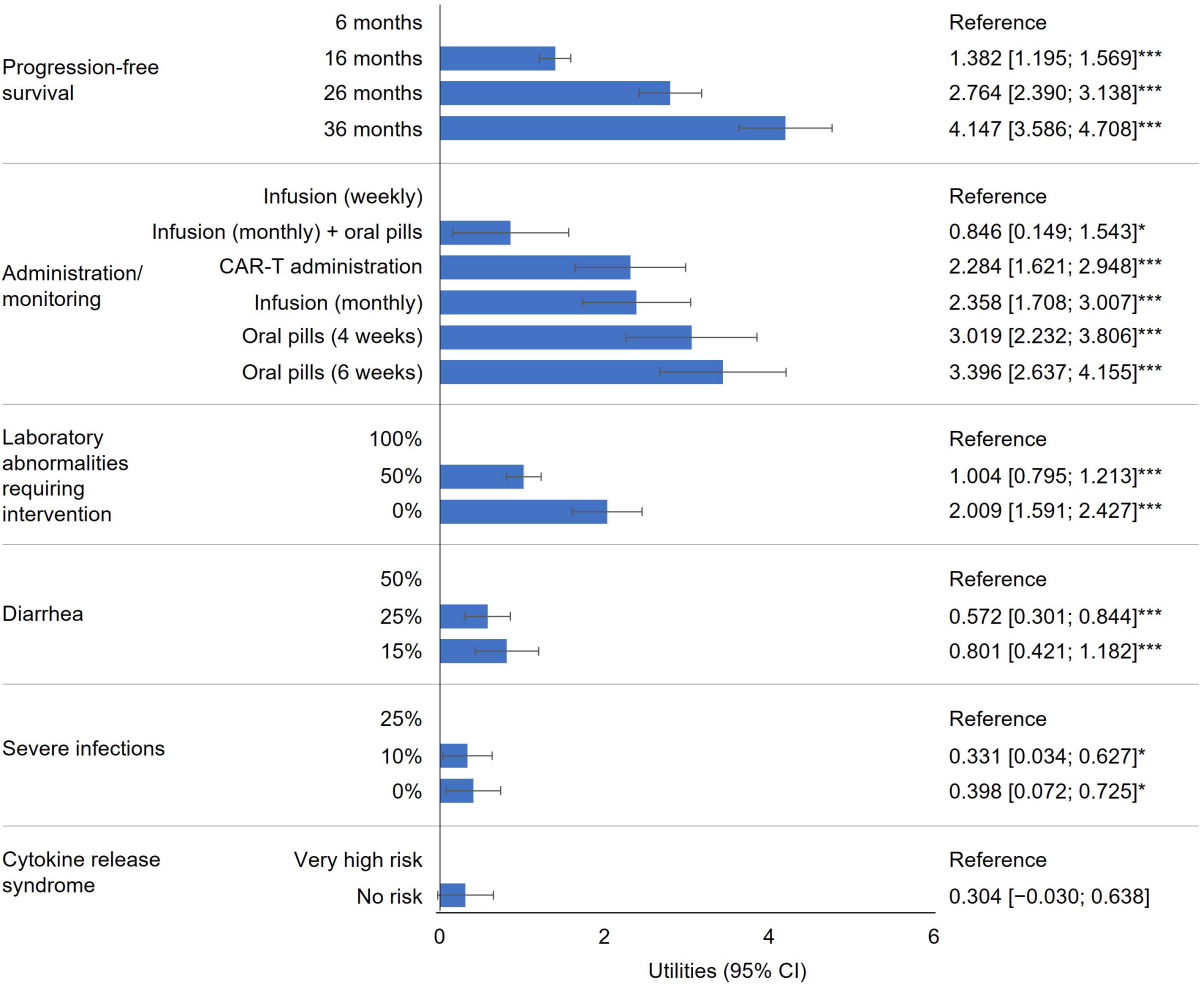
Averaged patient preferences (latent class results). *p < 0.05, **p < 0.01, ***p < 0.001 (vs. reference level for the corresponding attribute). CAR‐T, Chimeric Antigen Receptor T‐cell.

**FIGURE 3:**
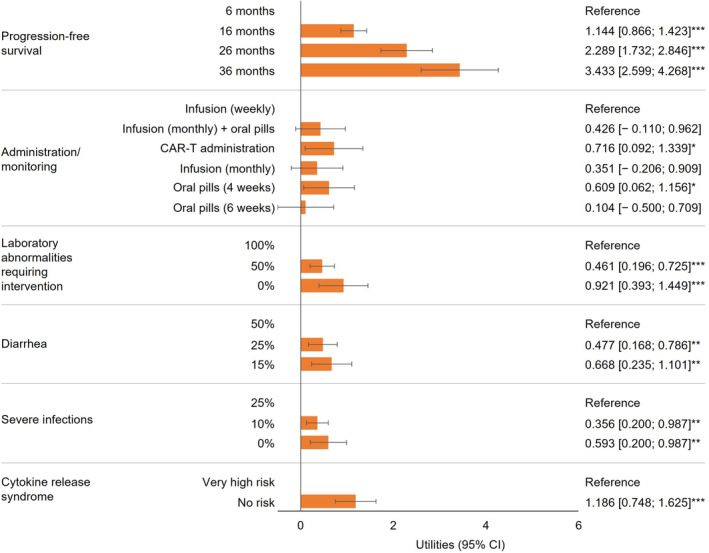
Averaged physician preferences (mixed logit results). *p < 0.05, **p < 0.01, ***p < 0.001 (vs. reference level for the corresponding attribute). CAR‐T, Chimeric Antigen Receptor T‐cell; CI, confidence interval; MLE, maximum likelihood estimate; OS, overall survival; PFS, progression‐free survival; SE, standard error.

Model results for both groups of participants confirmed that preference effects were in the expected direction for naturally ordered attributes: on average, both patients and physicians preferred to increase efficacy and avoid risks (Figure 2, Figure 3, Tables S2, S3). There was little impact of patients' observable characteristics on treatment preferences (Table S4), so patients' preferences are presented in this paper as an average of latent class logit model results across the sample. For patients, for all attributes except CRS, there was a statistically significant utility estimate for at least one attribute level versus the reference. For physicians, for all attributes, there was a statistically significant utility estimate for at least one attribute level versus the reference. These results suggest that patients considered most attributes when making their choices and physicians considered all attributes when making theirs, confirming the face validity of the DCE.

**FIGURE 4:**
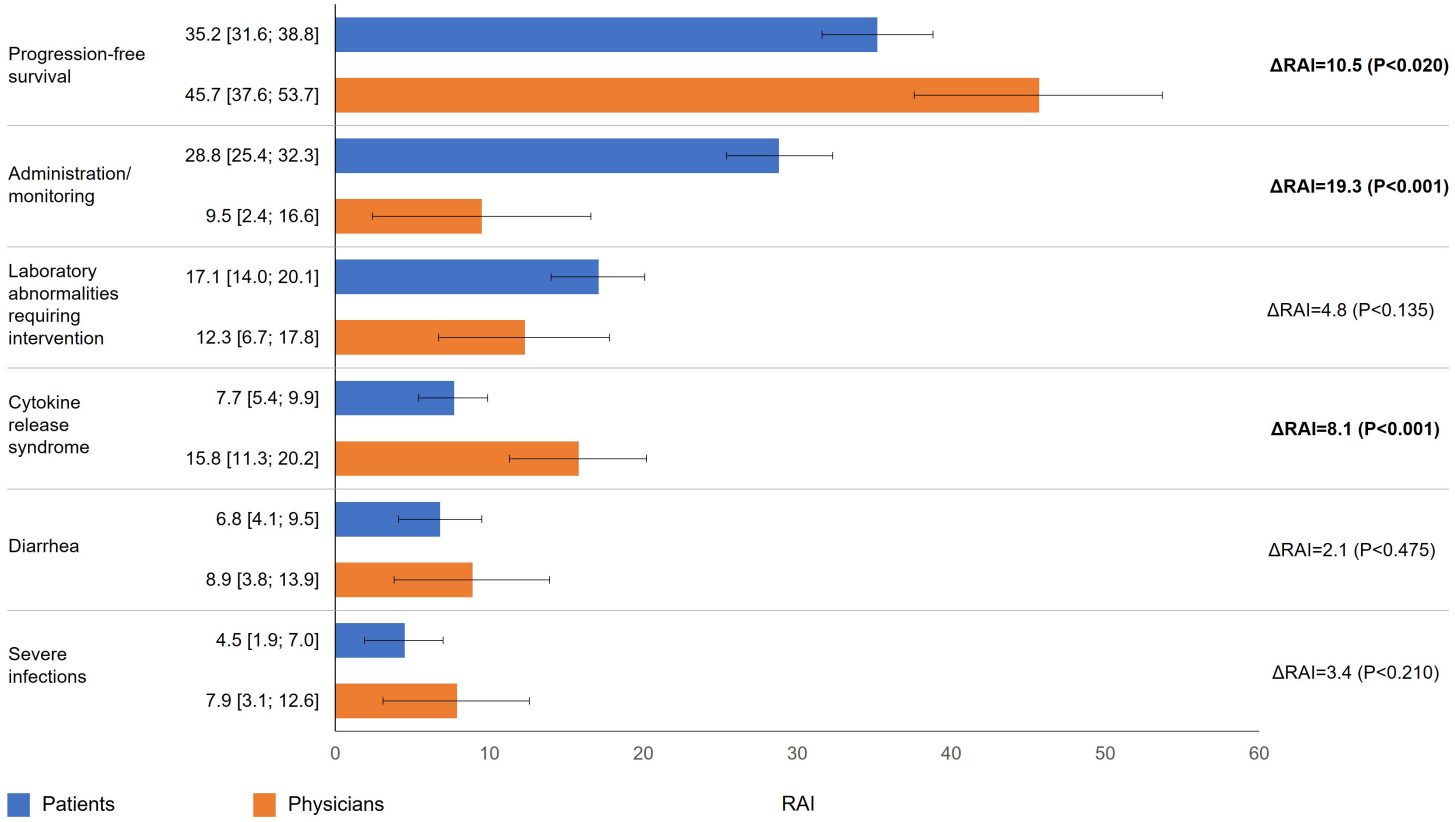
Relative attribute importance scores for patients and physicians. Error bars denote 95% confidence intervals, ΔRAI in bold denote statistically significant differences. ΔRAI, difference in relative attribute importance between patients and physicians; RAI, relative attribute importance.

PFS was the most important treatment attribute to both patients (RAI: 35.2% [confidence interval (CI): 31.6%–38.8%]) and physicians (for whom almost half of the treatment decision‐making was based on PFS; RAI: 45.7% [CI: 37.6%–53.7%]). However, physicians placed greater relative importance than patients on PFS than patients (ΔRAI: 10.5%; *p* = 0.020). After PFS, the most important treatment attributes for patients were administration/monitoring (RAI: 28.8% [CI: 25.4%–32.3%]) and laboratory abnormalities requiring intervention (RAI: 17.1% [CI: 14.0%–20.1%]). Administration/monitoring was more important to patients than it was to physicians (ΔRAI: 19.3%; *p* < 0.001) (Figure 4). Notably, both groups had slightly different preferences within administration/monitoring, with patients preferring daily oral pills with monitoring every 6 weeks and physicians preferring CAR‐T administration (Figure 2, Figure 3).

For physicians, the most important treatment attributes after PFS were CRS (RAI: 15.8% [CI: 11.3%–20.2%]) and laboratory abnormalities requiring intervention (RAI: 12.3% [CI: 6.7%–17.8%]). Avoiding CRS was more important to physicians than it was to patients (ΔRAI: 8.1%; *p* < 0.001), but physicians and patients attributed similar relative importance to laboratory abnormalities requiring intervention (ΔRAI: 4.8%; *p* = 0.135). Both groups also attributed similar lower relative importance to diarrhea (ΔRAI: 2.1%; *p* = 0.475) and severe infections (ΔRAI: 3.4%; *p* = 0.210) (Figure 4).

### 3.4 Willingness to trade‐off between attributes

**FIGURE 5:**
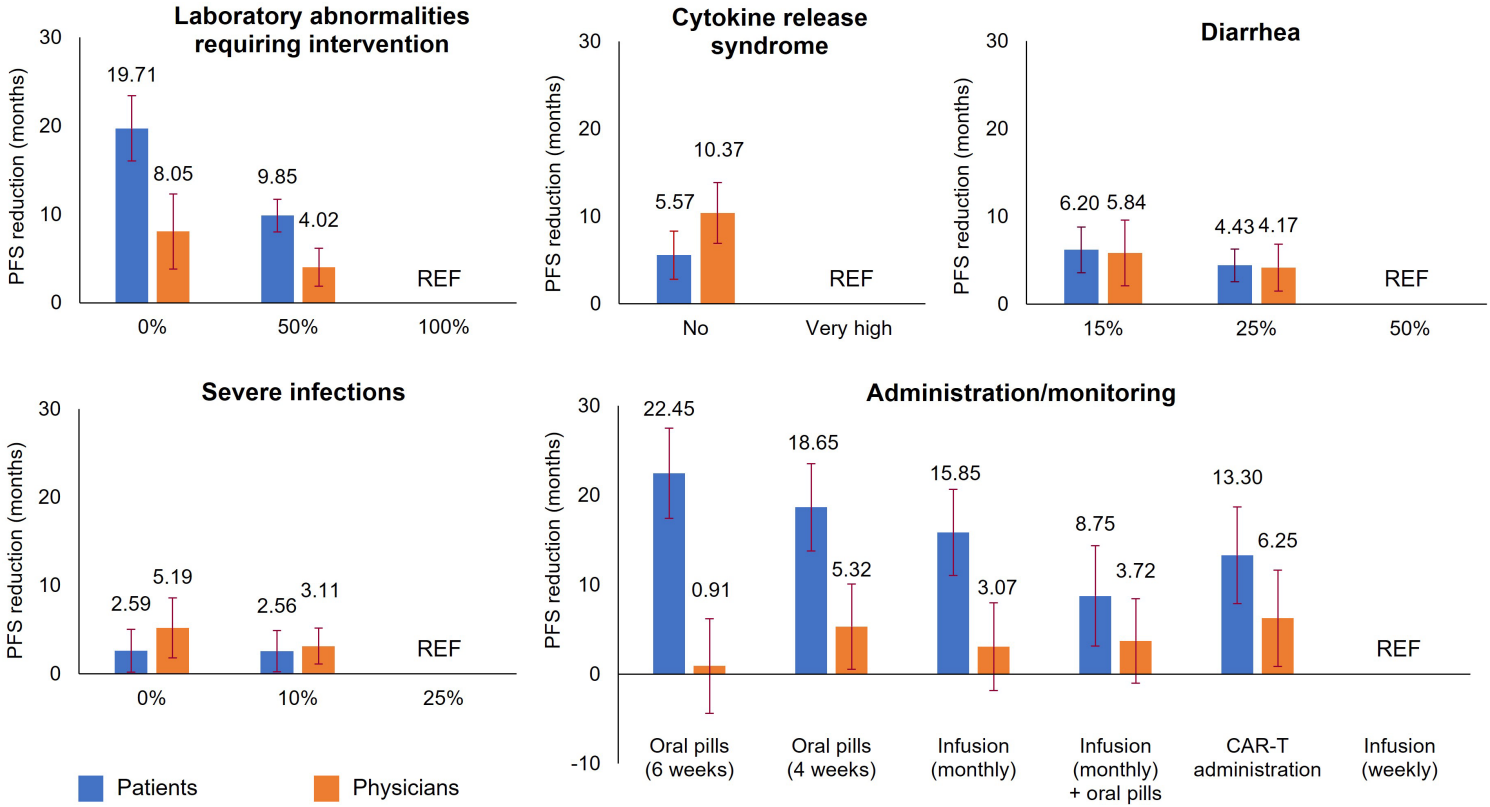
Patients' and physicians' willingness to trade off PFS for benefits in other treatment attributes. Numbers on bars denote the mean PFS reduction that each group of participants would be willing to tolerate in each scenario. Error bars denote 95% confidence intervals; “REF” denotes reference categories. 95% confidence intervals that cross the x‐axis indicate that there is not a significant willingness to trade‐off PFS for changes in another attribute to the relevant level (e.g. infusion [monthly] + oral pills for physicians). CAR‐T, chimeric antigen receptor T‐cell; PFS, progression‐free survival.

Both patients and physicians were willing to accept a treatment with a shorter PFS for improvements in other treatment attributes (Figure 5). For a treatment with a 50% risk of laboratory abnormalities requiring intervention (vs. one with 100% risk), patients would accept a 9.9‐month shorter PFS and physicians would accept a 4.0‐month shorter PFS. For a treatment with no risk of CRS (vs. one with very high risk), patients would accept an average reduction of 5.6 months in PFS, compared with 10.4 months for physicians. For a treatment with a 15% risk of diarrhea (vs. one with 50% risk), patients would accept an average reduction of 6.2 months in PFS, compared with 5.8 months for physicians. Lastly, for a treatment with no risk of severe infections (vs. one with 25% risk), patients would accept an average reduction of 2.6 in PFS, compared with 5.2 months for physicians.

Participants would also be willing to tolerate shorter PFS for a treatment with a preferred mode of administration/monitoring. Specifically, patients would accept a treatment with a 22.5‐month shorter PFS if it had daily oral administration with monitoring every 6 weeks instead of a treatment requiring weekly infusions. By contrast, physicians would be willing to tolerate a 6.3‐month shorter PFS for CAR‐T administration instead of weekly infusions.

## 4 DISCUSSION

The available treatment options for R/R FL have significantly expanded in the last decade.^4^ The increased number of options and the lack of a clear standard of care make treatment decisions more complex,^4^, ^12^ as trade‐offs are required when deciding which treatment to use. Patients, whose involvement in clinical decision‐making is increasingly encouraged,^9^, ^10^, ^15^ have been shown to make trade‐offs between treatment attributes.^13^ However, little is known about how patients' and physicians' preferences and trade‐offs might differ in R/R FL.

This DCE study elicited treatment preferences of patients with R/R FL as well as physicians treating FL. While physicians placed a significantly greater importance than patients on increasing PFS, PFS was the most important consideration for both groups. This is consistent with findings from previous studies, which also highlighted that increasing PFS was key to both patients and physicians.^13^, ^16^ The current study also explored the extent to which participants would be willing to accept a treatment that provides lower efficacy than another treatment option if it was more tolerable and/or had a preferred mode of administration. Both patients and physicians were willing to accept a treatment with a shorter PFS benefit if it was associated with a sufficiently lower risk of side effects or more convenient administration/monitoring; therefore, while efficacy is key, treatment preferences are also influenced by safety and administration. Specifically, patients would trade off 9.9 months of PFS for a treatment with a 50% lower risk of laboratory abnormalities requiring intervention, and physicians would trade off 10.4 months of PFS for a treatment with no risk of CRS.

Our study found that oral treatments are attractive to patients. Specifically, patients preferred all‐oral treatments and would be willing to accept a substantial reduction of PFS for this option compared with weekly infusions. Given patients' preference for oral administration, these findings encourage consideration of oral treatment options, such as tazemetostat or the recently approved zanubrutinib, for use in R/R FL.^27^, ^28^ Notably, the administration method used may be more important to patients with R/R FL than with other conditions, due to R/R FL being a chronic disease that patients live with for many years.^4^, ^5^ Although physicians preferred CAR‐T administration, on average, they also preferred treatments with oral administration and monitoring every 6 weeks to intravenous administration (although this effect did not reach statistical significance, potentially due to lack of statistical power and variability among responses). Physicians' preference towards oral versus intravenous administration is in agreement with a recent study of 300 physicians from six countries.^16^ In a previous study of 81 patients and 48 physicians in Canada, treatment administration did not affect preferences.^13^ However, in that study, all possible administration options were intravenous, and neither an all‐oral or CAR‐T alternative were included.^13^ Like that study, our work found that physician preferences were not statistically different for the different frequencies of intravenous administration; for patients, we did find that monthly infusions with or without oral pills were preferred to weekly infusions.

Notably, the present study demonstrates that the priorities of patients and physicians may differ. After increasing PFS, patients placed the greatest relative weight on achieving a preferred mode of administration/monitoring. This aspect was approximately three times more important to patients than it was to physicians. By contrast, after increasing PFS, physicians prioritized avoiding side effects. Patients and physicians also differed in which side effects they thought were most important to avoid. Laboratory abnormalities requiring intervention were the most important to patients, while CRS was the most important to physicians. Together, these differences between patients and physicians highlight the need for shared decision‐making to ensure that physicians' treatment selection incorporates a patient's priorities and preferences. Of note, while significant preference heterogeneity among patients was identified, this was largely unattributable to observable characteristics, with only health literacy, sex at birth, and response time found to influence the grouping of patients with similar preferences. Therefore, no assumptions can be made about the preferences of different groups of patients and shared decision‐making discussions are required on a case‐by‐case basis. Furthermore, given varied levels of health literacy and the complexity of oncology terminology, patient‐facing materials and discussions should aim to simplify language as much as possible.

Potential limitations include sample selection bias, whereby selected participants may not be representative of the general target population, although here it was minimized by using physician referrals. However, there may still be differences in preferences between those who are invited and consent to take part and those who either are not invited or did not consent to take part in the study. Hypothetical bias may also occur in DCEs, whereby participants' choices in hypothetical settings may differ from the choices they would make in real‐life situations.^14^ To reduce this bias and ensure the DCE was clinically accurate, understandable, and relevant, the development of this study extensively incorporated feedback from key stakeholders, including an expert oncologist, the Lymphoma Research Foundation, and the target population (via pilot interviews). Another limitation was that the study used patients' self‐reported clinical history rather than medical records or clinicians providing clinical information; this approach was used to minimize access to personally identifiable information. Further, attributes and levels were not identified through separate qualitative research but through a targeted review of previous quantitative and qualitative studies and clinical data for R/R FL treatments. The literature search was broader than FL including all NHL, due to the paucity of preference research in FL, although this is unlikely to have impacted the results because similar attributes were also identified and included in previous R/R FL preference studies.^13^, ^16^

## 5 CONCLUSIONS

In R/R FL, increasing PFS was the most important attribute to patients and physicians, although significantly more so for physicians. The differences in the preference for oral treatment between both groups also highlighted the need for shared decision‐making during treatment selection: in this chronic disease, patients prioritized the convenience of oral administration and would be willing to accept a substantial reduction in PFS for this option. Both patients and physicians would be willing to accept a treatment with shorter PFS if adequately compensated with lower risks of side effects. While the results of this study should be interpreted within the context of the study sample, physicians could use these findings as a starting point for discussions with patients about their priorities during this chronic illness, ensuring that treatment decisions align with individual patient preferences.

## AUTHOR CONTRIBUTIONS

**Caitlin Thomas:** Conceptualization (equal); investigation (equal); methodology (equal); supervision (equal); visualization (equal). **Kevin Marsh:** Conceptualization (equal); investigation (equal); methodology (equal); project administration (equal); supervision (equal); visualization (equal). **Myrto Trapali:** Investigation (equal); validation (equal). **Nicolas Krucien:** Formal analysis (equal). **Gavin Worth:** Supervision (equal). **Paul Cockrum:** Data curation (equal); formal analysis (equal); investigation (equal); methodology (equal); project administration (equal); supervision (equal); validation (equal); visualization (equal). **Debra Lycett:** Conceptualization (equal); supervision (equal).

## FUNDING INFORMATION

This study was funded by Ipsen.

## ETHICS STATEMENT

The study was approved by Salus institutional review board (formerly Ethical & Independent; study number: 21809–01) in June 2021 and an amendment was approved in August 2021.

## Supporting information


Table S1‐S4.

## Data Availability

Qualified researchers may request access to patient‐level study data that underlie the results reported in this publication. Additional relevant study documents, including the clinical study report, study protocol with any amendments, annotated case report form, statistical analysis plan and dataset specifications may also be made available. Patient level data will be anonymized, and study documents will be redacted to protect the privacy of study participants. Where applicable, data from eligible studies are available 6 months after the studied medicine and indication have been approved in the US and EU or after the primary manuscript describing the results has been accepted for publication, whichever is later. Further details on Ipsen's sharing criteria, eligible studies and process for sharing are available here (https://vivli.org/members/ourmembers/). Any requests should be submitted to www.vivli.org for assessment by an independent scientific review board.
