# Supplementary material for: Preferences of patients and physicians in the United States for relapsed/refractory follicular lymphoma treatments
Source: Cancer Med. 2024 Oct 12;13(19):e70177. doi: 10.1002/cam4.70177 (PMC11470200; doi:10.1002/cam4.70177)
Supplement: Supplementary file 1 — Table S1‐S4. [file CAM4-13-e70177-s001.docx]

## Supplementary Table 1. Definitions of treatment attributes.

| **Attribute Groups** | **Physicians** | **Patients** |
| --- | --- | --- |
| Administration and monitoring | Administration and monitoring  This study includes treatments with the following types of administration. The frequency of monitoring is associated with the type of treatment given.   - Oral pills - IV infusions - Combinations of oral pills and IV infusions - CAR-T therapy: - One-time treatment with several steps, which takes around one month. - Blood is taken from the patient over two to three hours (leukapheresis) and is sent off to a laboratory for reprogramming. - A few days prior to reinfusion, patients are given bridging chemotherapy as an inpatient in a hospital familiar with administering CAR-T therapy. - Reprogrammed blood is reinfused as an inpatient in the hospital familiar with administering CAR-T therapy. - After the reinfusion, they would need to stay in the hospital familiar with administering CAR-T therapy for seven days following reinfusion so that they can be closely monitored. - Upon leaving the hospital, they would need to stay within proximity (within two hours) to the hospital where they received treatment for four weeks in case they experience severe side effects that need urgent treatment. This may require them to book alternative accommodation for this period. | Administration and check-ups  There are a variety of treatments available or being developed for FL, with different ways of administering treatment. Some treatments are oral pills, which can be taken at home; others require one-hour intravenous (IV; into the vein) infusions at hospital, as an outpatient; and some require a combination of both oral pills at home and one-hour IV infusions at hospital, as an outpatient. The frequency of the IV infusions also varies from weekly to monthly.  One alternative treatment is a blood training treatment, also known as CAR-T therapy. This is a one-time treatment that involves several steps over a period of one month.   - Your blood is taken at a hospital familiar with administering CAR-T therapy. You will attend as an outpatient over two to three hours. - Your blood will be sent to a laboratory and is “trained” to fight the cancer cells—a process that takes a few weeks. - You are then reinfused with the “trained” blood as an inpatient at the hospital.   - To prepare for this, you will need to stay in the hospital for a few days to be given additional chemotherapy, prior to reinfusion—which may make you feel unwell.   - After the reinfusion, you will need to stay in the hospital where you received treatment for another seven days (for monitoring).   - Upon leaving the hospital, you will need to stay close (within two hours) to the hospital for four weeks in case you have severe side effects that need urgent treatment. This may require you to book alternative accommodations for this period.   All treatments have some risk of side effects, which are described later in the survey. Although the level of risk varies between treatments, you will need to visit your healthcare provider for check-ups. This will involve blood tests, which take approximately 30 minutes. The frequency of check-ups required depends on the treatment you are taking. |
| Benefit | PFS and OS  The length of PFS following treatment will vary from 6 months to 3 years. Regardless of PFS, OS will be 10 years for all treatments. | Time without cancer growth  Treatments aim to shrink or control the cancer. The treatments will differ in the time period for which they stop your cancer growing. During this period, you may be less worried about your cancer and have fewer disease-related symptoms. If your cancer starts to grow again, your doctor may change your treatment to try to get it back under control.  Time alive  Regardless of difference in time without cancer growth, there are no differences in the length of time you will be alive (overall survival) following treatments included in this study. Following all treatments, you will live for 10 years. |
| Side effects | Risk of diarrhea  FL treatments may cause diarrhea, but the risk of experiencing diarrhea depends on the treatment patients are given. This risk varies from 15% to 50%.  Patients experiencing diarrhea will have at least two to three loose stools per day. Diarrhea often means frequent trips to the bathroom. This can cause discomforts like itching, burning and pain during bowel movements, and can also impact on a patient’s ability to do daily activities. Examples of daily activities include productivity at work or home, going to social events, shopping, caring for children and relatives, and enjoying leisure activities.  In around one third of cases, diarrhea is severe, and patients would have six or more watery stools that require outpatient IV hydration, an emergency room visit, or hospitalization. | Number of people out of 100 on treatment who have diarrhea  FL treatments may cause diarrhea, but the chance (risk) of experiencing diarrhea depends on the treatment you are given. This chance varies from 15 out of 100 people (15%) to 50 out of 100 (50%) people.  If you have diarrhea, you will have at least two to three loose stools per day. Diarrhea often means frequent trips to the bathroom. This can cause discomforts like itching, burning and pain during bowel movements, and can also impact your ability to do daily activities. Examples of daily activities include productivity at work or home, going to social events, shopping, caring for children and relatives, and enjoying leisure activities.  In some cases (around one in three people), diarrhea is severe, and you may have six or more watery stools that require outpatient IV hydration, an emergency room visit, or hospitalization. |
|  | Risk of severe infections  FL treatments may cause severe infections, such as lung infections or pneumonia. If patients experience severe infections, they will need IV antibiotics and/or hospitalization. In rare cases, the severe infections can be life-threatening. | Number of people out of 100 on treatment who have severe infections.  FL treatments may cause severe infections, such as lung infections or pneumonia. If you have severe infections, you will feel unwell and will need IV antibiotics and/or hospitalization. In rare cases, the severe infections can be life-threatening. |
|  | Risk of severe laboratory abnormalities requiring intervention  Some treatments have a higher risk of laboratory abnormalities (neutropenia, increase in alanine aminotransferase, increase in aspartate amino transferase, hypokalemia, anemia, thrombocytopenia) that requires intervention. This may involve additional treatment, such as blood transfusions, or hospitalization. In rare cases, severe laboratory abnormalities can be life-threatening. | Number of people out of 100 on treatment who have blood test results requiring intervention.  For all treatments, you will require check-ups that involve blood tests. Your doctor will look at the blood test results to check how the treatment is working, and whether the treatment is producing any side effects.  Some treatments have a higher risk of producing blood test results that require intervention from your doctor to address a problem. This may involve additional treatment, such as blood transfusions or hospitalization to correct any problems with your blood results. In rare cases, severe blood results can be life-threatening. |

|  | CRS  Certain types of treatments have a very high risk of CRS, which is a systemic inflammatory response caused by a large, rapid release of cytokines into the blood from immune cells. CRS can be harmful and interfere with bodily functions, causing organ failure and death in severe cases.  In this study, you will be shown some treatments that have a very high risk of CRS and others that do not. If a treatment has a very high risk of CRS, of 100 patients on the treatment, the experience of CRS is summarized below:   - 5 out of 100 people (5%) will not experience CRS when using a treatment that has a very high risk of CRS. - For 80 out of 100 people (80%), CRS is not severe, and will involve side effects such as fever, chills, fatigue, weakness, loss of appetite, nausea, vomiting, diarrhea, headache, joint or muscle aches, or skin rash. Patients may be given oxygen if they have difficulty breathing, or IV immunosuppressants to treat CRS. - For 10 out of 100 people (10%), CRS results in severe side effects, such as damage to the heart, brain, lungs, kidney, and/or liver. This is treated with IV steroids, as well as oxygen and immunosuppressants. In some cases, patients with severe side effects need to be put on a ventilator to support their breathing or given continuous veno-venous hemodialysis for acute renal failure. - For five out of 100 people (5%), CRS is fatal. | Inflammation  Some treatments have a very high risk of inflammation, whereas others do not. Inflammation can be harmful and interfere with bodily functions, causing organ failure and death in severe cases.  In this study, you will be shown some treatments that have a very high risk of inflammation and others that do not. If a treatment has a very high risk of inflammation, of 100 patients on the treatment, the inflammation experience is summarized below:   - 5 out of 100 people (5%) do not have inflammation, or side effects from inflammation, when using a treatment that has a very high risk of inflammation. - For 80 out of 100 people (80%), inflammation is not severe, and will involve side effects such as fever, chills, fatigue, weakness, loss of appetite, nausea, vomiting, diarrhea, headache, joint or muscle aches, or skin rash. You may be given oxygen if you have difficulty breathing, or IV immunosuppressants to treat the inflammation. - For 10 out of 100 people (10%), inflammation results in severe side effects, such as damage to the heart, brain, lungs, kidney, and/or liver. This is treated with IV steroids, as well as oxygen and immunosuppressants. In some cases, patients with severe side effects need to be put on a ventilator to support their breathing or given dialysis for damaged kidneys. - For five out of 100 people (5%), inflammation is fatal. |
| --- | --- | --- |

Abbreviations: CAR-T, chimeric antigen receptor T-cell; CRS, cytokine release syndrome; IV, intravenous; OS, overall survival; PFS, progression-free survival.

## Supplementary Table 2. Marginal utilities for patients (averaged latent class results).

| **Attribute** | **Patients (N=200)^†^** | |
| --- | --- | --- |
|  | **Marginal utilities (SE)** | **95% CI** |
| Alternative specific constant | | |
| Treatment A | Reference | |
| Treatment B | 0.1081 (0.1364) | −0.1591, 0.3754 |
| Neither A nor B | 3.8933 (0.4417)*** | 3.0276, 4.7591 |
| Administration and monitoring | | |
| Oral pills daily, monitoring every 6 weeks | 3.3959 (0.3870)*** | 2.6373, 4.1545 |
| Oral pills daily, monitoring every 4 weeks | 3.0192 (0.4015)*** | 2.2323, 3.8061 |
| IV infusion every month, monitoring during infusion appointment | 2.3578 (0.3314)*** | 1.7082, 3.0074 |
| IV infusion every month and daily oral pill, monitoring during infusion appointment | 0.8459 (0.3556)* | 0.1488, 1.5429 |
| IV infusion every week, monitoring during infusion appointment | Reference | |
| CAR-T therapy. Takes 1 month – one-time treatment. Inpatient in hospital for 7 days after treatment. Must stay near hospital for 4 weeks for monitoring | 2.2843 (0.3386)*** | 1.6206, 2.9479 |
| PFS^‡^ | | |
| 3 years | 4.1466 (0.2862)*** | 3.5857, 4.7076 |
| 2 years and 2 months | 2.7644 (0.1908)*** | 2.3904, 3.1384 |
| 1 year and 4 months | 1.3822 (0.0954)*** | 1.1952, 1.5692 |
| 6 months | Reference | |
| Risk of diarrhea | | |
| 15% (15 out of 100 people) | 0.8015 (0.1941)*** | 0.4210, 1.1820 |
| 25% (25 out of 100 people) | 0.5725 (0.1387)*** | 0.3007, 0.8443 |
| 50% (50 out of 100 people) | Reference | |
| Risk of severe infections | | |
| 0% (0 out of 100 people) | 0.3983 (0.1665)* | 0.0720, 0.7247 |
| 10% (10 out of 100 people) | 0.3305 (0.1513)* | 0.0339, 0.6272 |
| 25% (25 out of 100 people) | Reference | |
| Risk of severe lab abnormalities requiring intervention | | |
| 0% (0 out of 100 people) | 2.0087 (0.2133)*** | 1.5906, 2.4267 |
| 50% (50 out of 100 people) | 1.0043 (0.1066)*** | 0.7953, 1.2134 |
| 100% (100 out of 100 people) | Reference | |
| CRS | | |
| No risk | 0.3040 (0.1706) | −0.0302, 0.6383 |
| Very high risk | Reference | |

* *p*<0.05, ** *p*<0.01, *** *p*<0.001 (versus reference level for the corresponding attribute).

^†^Model information: Respondents = 200; observations = 1600; parameters = 41; log-likelihood = −1203.1; BIC= 2708.6.

^‡^Assuming 10-year overall survival.

Abbreviations: BIC, Bayesian information criteria; CAR-T, chimeric antigen receptor T-cell; CI, confidence interval; CRS, cytokine release syndrome; IV, intravenous; PFS, progression-free survival; SE, standard error.

## Supplementary Table 3. Marginal utilities for physicians (mixed logit results).

| **Attribute** | **Physicians (N=151)**^†^ | | | |
| --- | --- | --- | --- | --- |
|  | **Average** | | **Dispersion (SD)** | |
|  | **Marginal utilities (SE)** | **95% CI** | **Marginal utilities (SE)** | **95% CI** |
| Alternative specific constant | | | | |
| Treatment A | Reference | | | |
| Treatment B | −0.391 (0.163)* | −0.711, −0.070 | 0.856 (0.208)*** | 0.449, 1.264 |
| Neither A nor B | 0.507 (0.397) | −0.271, 1.284 | 2.259 (0.387)*** | 1.500, 3.018 |
| Administration and monitoring | | | | |
| Oral pills daily, monitoring every 6 weeks | 0.104 (0.308) | −0.500, 0.709 | 1.237 (0.442)** | 0.370, 2.103 |
| Oral pills daily, monitoring every 4 weeks | 0.609 (0.279)* | 0.062, 1.156 | 0.292 (0.486) | −0.660, 1.243 |
| IV infusion every month, monitoring during infusion appointment | 0.351 (0.284) | −0.206, 0.909 | 0.845 (0.431)* | 0.001, 1.689 |
| IV infusion every month and daily oral pill, monitoring during infusion appointment | 0.426 (0.274) | −0.110, 0.962 | 0.592 (0.518) | −0.423, 1.606 |
| IV infusion every week, monitoring during infusion appointment | Reference | | | |
| CAR-T therapy. Takes 1 month – one-time treatment. Inpatient in hospital for 7 days after treatment. Must stay near hospital for  4 weeks for monitoring | 0.716 (0.318)* | 0.092, 1.339 | 0.829 (0.521) | −0.193, 1.851 |
| PFS^‡^ | | | | |
| 3 years PFS | 3.433 (0.426)*** | 2.599, 4.268 | 2.499 (0.407)*** | 1.702, 3.296 |
| 2 years and 2 months PFS | 2.289 (0.284)*** | 1.732, 2.846 | 1.666 (0.271)*** | 1.134, 2.197 |
| 1 year and 4 months PFS | 1.144 (0.142)*** | 0.866, 1.423 | 0.833 (0.136)*** | 0.567, 1.099 |
| 6 months PFS | Reference | | | |

| Risk of diarrhea | | | | |
| --- | --- | --- | --- | --- |
| 15% (15 out of 100 people) | 0.668 (0.221)** | 0.235, 1.101 | 0.975 (0.318)** | 0.353, 1.597 |
| 25% (25 out of 100 people) | 0.477 (0.158)** | 0.168, 0.786 | 0.696 (0.227)** | 0.252, 1.141 |
| 50% (50 out of 100 people) | Reference | | | |
| Risk of severe infections | | | | |
| 0% (0 out of 100 people) | 0.593 (0.201)** | 0.200, 0.987 | 1.107 (0.298)*** | 0.524, 1.691 |
| 10% (10 out of 100 people) | 0.356 (0.120)** | 0.120, 0.592 | 0.664 (0.179)*** | 0.314, 1.015 |
| 25% (25 out of 100 people) | Reference | | | |
| Risk of severe lab abnormalities requiring intervention | | | | |
| 0% (0 out of 100 people) | 0.921 (0.270)*** | 0.393, 1.449 | 1.653 (0.305)*** | 1.055, 2.251 |
| 50% (50 out of 100 people) | 0.461 (0.135)*** | 0.196, 0.725 | 0.827 (0.153)*** | 0.528, 1.126 |
| 100% (100 out of 100 people) | Reference | | | |
| CRS | | | | |
| No risk | 1.186 (0.224)*** | 0.748, 1.625 | 1.432 (0.252)*** | 0.938, 1.926 |
| Very high risk | Reference | | | |

* *p*<0.05, ** *p*<0.01, *** *p*<0.001 (versus reference level for the corresponding attribute).

^†^Model information: Individuals = 151; observations = 1208; parameters = 24; log-likelihood = −981.1; BIC= 2132.5.

^‡^Assuming 10-year overall survival.

Abbreviations: BIC, Bayesian information criteria; CAR-T, chimeric antigen receptor T-cell; CI, confidence interval; CRS, cytokine release syndrome; IV, intravenous; SD, standard deviation; SE, standard error; PFS, progression-free survival.

## Supplementary Table 4. Class allocation results.

| **Characteristics** | **Levels** | **N (%)** | **Class 1 versus Class 2** | **Class 3 versus Class 2** | **Class 1 versus Class 3** |
| --- | --- | --- | --- | --- | --- |
| Constant | – | – | −1.118 (1.052) | −1.092 (1.058) | – |
| Age (group) | >65 years | 87 (44) | Reference | – | – |
|  | ≤65 years | 113 (56) | 0.089 (0.430) | −0.030 (0.442) | 0.12 (0.466) |
| Sex at birth | Male | 80 (40) | Reference | – | – |
|  | Female | 120 (60) | −0.320 (0.388) | −0.905 (0.391)* | 0.585 (0.413) |
| Remission status | In remission | 111 (56) | Reference | – | – |
|  | Not in remission | 89 (44) | 0.570 (0.630) | 0.981 (0.636) | −0.411 (0.661) |
| Line of therapy | 2 lines | 65 (32) | Reference | – | – |
|  | 3–4 lines | 135 (68) | 0.320 (0.634) | 0.719 (0.641) | −0.4 (0.685) |
| Ethnicity | Hispanic | 49 (24) | Reference | – | – |
|  | Not Hispanic | 151 (76) | −0.693 (0.466) | 0.337 (0.529) | −1.03 (0.543) |
| Racial Background | White | 80 (40) | Reference | – | – |
|  | Black or African American | 41 (20) | 0.425 (0.551) | 0.407 (0.541) | 0.018 (0.576) |
|  | Other | 79 (40) | 0.280 (0.443) | −0.024 (0.456) | 0.304 (0.498) |
| Level of education | High school or less | 77 (38) | Reference | – | – |
|  | Some college/ university or higher | 123 (62) | 0.358 (0.400) | 0.199 (0.408) | 0.159 (0.446) |
| Health literacy | Low (≤2) | 57 (28) | Reference | – | – |
|  | High (>2) | 143 (72) | 0.943 (0.466)* | 0.509 (0.453) | 0.433 (0.522) |
| General health | Mild limitations | 121 (60) | Reference | – | – |
|  | Severe limitations | 79 (40) | 0.405 (0.542) | 0.115 (0.560) | 0.29 (0.591) |
| DCE response time | <2 | 75 (38) | Reference | – | – |
|  | 2–5 | 54 (27) | −0.925 (0.480) | −0.149 (0.478) | −0.776 (0.505) |
|  | 5–15 | 71 (36) | −1.166 (0.432)** | −0.779 (0.461) | −0.387 (0.491) |
| Years since FL diagnosis | 0–3 years | 95 (48) | Reference | – | – |
|  | ≥4 years | 105 (52) | 0.049 (0.399) | −0.263 (0.411) | 0.312 (0.438) |
| ER visits relating to FL within the last 12 months | Yes | 65 (32) | Reference | – | – |
|  | No | 135 (68) | −0.145 (0.471) | 0.034 (0.493) | −0.179 (0.511) |
| Dependent family members | Yes | 25 (12) | Reference | – | – |
|  | No | 175 (88) | 0.501 (0.600) | −0.211 (0.577) | 0.712 (0.63) |

* *p*<0.05, ** *p*<0.01, *** *p*<0.001 (versus reference).

Class composition: Class 1 (n=62), Class 2 (n=85), Class 3 (n=53).

Abbreviations: DCE, discrete-choice experiment; ER, emergency room; FL, follicular lymphoma.
